# Semi-rigid ureteroscopic lithotripsy versus laparoscopic ureterolithotomy for large upper ureteral stones: a meta – analysis of randomized controlled trials

**DOI:** 10.1590/S1677-5538.IBJU.2015.0696

**Published:** 2016

**Authors:** Fabio C. M. Torricelli, Manoj Monga, Giovanni S. Marchini, Miguel Srougi, William C. Nahas, Eduardo Mazzucchi

**Affiliations:** 1 Departamento de Urologia do Hospital das Clínicas da Universidade de São Paulo Faculdade de Medicina São Paulo, SP, Brasil; 2 Stevan B. Streem Center for Endourology & Stone Disease; Glickman Urological & Kidney Institute, The Cleveland Clinic, Cleveland, OH, USA

**Keywords:** Laparoscopy, Lithotripsy, Ureter, Ureteroscopy, Urinary Calculi

## Abstract

**Introduction:**

To provide a systematic review and meta-analysis of randomized controlled trials (RCT) comparing semi-rigid ureteroscopic lithotripsy (URS) with laparoscopic ureterolithotomy (LU) for the treatment of the large proximal ureteral stone.

**Materials and methods:**

A systematic literature review was performed in June 2015 using the PubMed, Scopus, and Web of Science databases to identify relevant studies. Article selection proceeded according to the search strategy based on Preferred Reporting Items for Systematic Reviews and Meta-analysis criteria.

**Results:**

Six RCT including 646 patients were analyzed, 325 URS cases (50.3%) and 321 LU cases (49.7%). URS provided a significantly shorter operative time (weighted mean difference [WMD] = −31.26 min; 95%CI −46.88 to −15.64; p<0.0001) and length of hospital stay (WMD = −1.48 days; 95%CI −2.78 to −0.18; p=0.03) than LU. There were no significant differences in terms of overall complications (OR = 0.78; 95%CI 0.21-2.92; p=0.71) and major complications – Clavien ≥3 – (OR = 1.79; 95%CI 0.59-5.42; p=0.30). LU led to a significantly higher initial stone-free rate (OR = 8.65; 95%CI 4.18-17.91; p<0.00001) and final stone-free rate (OR = 6.41; 95%CI 2.24-18.32; p=0.0005) than URS. There was a significantly higher need for auxiliary procedures in URS cases (OR = 6.58; 95%CI 3.42-12.68; p<0.00001).

**Conclusions:**

Outcomes with LU for larger proximal ureteral calculi are favorable compared to semi-rigid URS and should be considered as a first-line alternative if flexible ureteroscopy is not available. Utilization of flexible ureteroscopy in conjunction with semi-rigid ureteroscopy may impact these outcomes, and deserves further systematic evaluation.

## INTRODUCTION

Ureteral stones may cause severe pain, lead to hydronephrosis and/or urinary tract infection, and ultimately may be the reason for renal function loss (1). Although small distal ureteral stones most commonly spontaneously pass through the ureter into the bladder, large proximal ureteral stones (>10mm) can take more than 2 – 3 weeks to pass all the way (2, 3). In a worst scenario, these stones can get impacted in the ureter, requiring surgical intervention.

Medical expulsive therapy using alpha-blockers (i.e. tamsulosin, alfuzosin) or calcium channel blockers (i.e. nifedipine) have been used for several years in the treatment of patients suffering from ureteral stone, reportedly resulting in a higher stone-free rate and a shorter time to stone expulsion when compared to placebo (4, 5). However, a recent multicenter, randomized, placebo-controlled trial has demonstrated different outcomes and questioned the role of medical expulsive therapy (6).

Thus, surgical intervention may be the best alternative for patients with refractory pain to analgesics, and early intervention may be considered for large proximal calculi that are unlikely to pass spontaneously. Although there is consensus that ureteroscopy is the most efficient treatment for patients with distal ureteral stones, there is a debate regarding large proximal ureteral stones (7, 8). AUA (American Urological Association) and EAU (European Association of Urology) have recommended ureteroscopic lithotripsy (URS) or shockwave lithotripsy (SWL) as first option, although percutaneous nephrolithotomy (PCNL) and laparoscopic ureterolithotomy (LU) may be suitable (3, 7–9).

Currently, there is a clear tendency of less SWL and more URS in the treatment of patient with urinary stones, even in developing countries (10). As flexible ureteroscopies are not available in all services, semi-rigid ureteroscopy has been used for treatment of ureteral stones in all locations, even for those in the proximal ureter. PCNL is a procedure with inherent high-risk of surgical complications, whereas LU has gained some popularity (11). Based on these concepts, in this meta-analysis we aimed to compare the outcomes from URS with those from LU for management of large proximal ureteral stones.

## MATERIAL AND METHODS

### Evidence acquisition - Literature search and study selection

**Figure 1:**
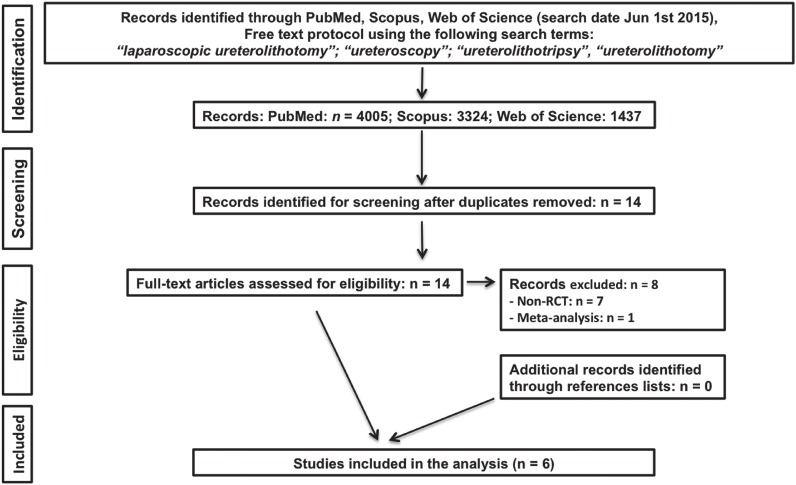
Preferred Reporting Items for Systematic Reviews and Meta-analysis flow of study selection.

A systematic literature review was performed in June 2015 using PubMed, Scopus, and Web of Science databases to identify relevant studies. Searches were restricted to publications in English and in the adult population. Separate searches were done with the following search terms: laparoscopic ureterolithotomy, ureteroscopy, ureterolithotripsy, ureterolithotomy. Article selection proceeded according to the search strategy based on Preferred Reporting Items for Systematic Reviews and Meta-analysis criteria (www.prismastatement.org) (Figure-1). Only studies comparing URS and LU were included for further screening. Only randomized controlled trials (RCT) were selected and included in the study. There is only one RCT comparing flexible ureteroscopy with laparoscopy (12), therefore the study focused on semi-rigid ureteroscopy. Cited references from the selected articles retrieved in the search were also assessed for significant papers. Conference abstracts were not included because sufficient detail for the study is not available in an abstract. Two independent reviewers completed this process, and all disagreements were resolved by their consensus.

### Study quality assessment

The level of evidence was rated for each included study according to the criteria provided by the Center for Evidence-Based Medicine in Oxford, UK (13). The methodological quality of RCT was assessed using the Jadad scale, which goes from 0 to 5 points (14).

#### Statistical analysis

A meta-analysis was performed to assess the overall outcomes of URS compared with LU. Extracted data for the analysis included operative time, length of hospital stay, need for auxiliary procedures, and postoperative complication and stone-free rates. Complications were scored according Clavien classification (15). Residual fragments or stone migration during URS or LU were not considered as complications, because these findings were evaluated by the stone-free rate. Initial stone-free rate was evaluated immediately after surgery, whereas final stone-free rate was evaluated after auxiliary procedures or spontaneous passage at least 3 weeks (3 weeks to 1 year) after the first procedure. Odds ratio (OR) was used for binary variables, and mean difference or standardized mean difference was used for the continuous parameters. For studies presenting continuous data as means and range, standard deviations were calculated using the methodology described by Hozo and associates (16). Pooled estimates were calculated with the fixed-effect model (Mantel-Haenszel method) if no significant heterogeneity was detected; otherwise, the random-effect model (Der Simonian-Laird method) was used. The pooled effects were determined by the z test, and p<0.05 was considered statistically significant. The Cochrane chi-square test and inconsistency (I^2^) were used to evaluate the heterogeneity among studies. Data analysis was performed with Review Manager software (RevMan v.5.1, Cochrane Collaboration, Oxford, UK).

## RESULTS

### Evidence synthesis - Study characteristics

Six RCT including 646 patients were selected for the analysis, 325 URS cases (50.3%) and 321 LU cases (49.7%) Table-1 (17–22). There were no differences regarding age (40.9 vs. 41.0 years, respectively), gender (61.2% vs. 62.5% male, respectively), stone size (13.6 vs. 18.2mm, respectively) and laterality of the procedure (49.7% vs. 52.4%, respectively) between the groups. The methodological quality of included studies was medium, as they scored 2 of 5 points (17, 19, 20, 22) or 3 of 5 points (18, 21) in Jadad scale; as surgical blinded studies are hard to be conducted, two points of Jadad scale were lost in all studies (Table-2).

**Table 1:** Demographic data.

	URS	LU	p-value
Number of cases (n)	325	321	-
Age (mean, ± SD)	40.9 ± 5.1	41.0 ± 4.7	0.936
Gender (male, %)	61.20%	62.60%	0.746
Stone size (mean, ± SD)	13.6 ± 7.8	18.2 ± 4.2	0.298
Side (right, %)	49.70%	52.40%	0.604

**URS** = ureteroscopic lithotripsy; **LU** = laparoscopic ureterolithotomy; **SD** = standard deviation

**Table 2:** Ureteroscoplc lithotripsy versus laparoscopic ureterolithotomy: summary data of randomized controlled trials.

Study	N of cases		Study period	Study Design	Level of evidence	Inclusion criteria (stone size)	Energy source of URS	LU access	Control imaging exam	Quality score*
	URS	LU								
Basiri et al.	50	50	2004-2006	RCT	2b	≥15 mm	Pneumatic or laser	Transperitoneal	KUB and USG	2
Lopes Neto et al.	16	15	2008-2010	RCT	2b	≥10mm	Pneumatic	10 trans and 5 retroperitoneal	KUB or CT	3
Fang et al.	25	25	2008-2010	RCT	2b	≥10mm	Laser	Retroperitoneal	KUB	2
Shao et al.	139	136	2009-2013	RCT	2b	≥12 mm	Laser	Retroperitoneal	NA	2
Kumar et al.	50	50	2010-2012	RCT	2b	≥20mm	Laser	Transperitoneal	CT	3
Liu et al.	45	45	2011-2013	RCT	2b	NA	Laser	Retroperitoneal	KUB	2

**URS** = ureteroscopic lithotripsy; **LU** = laparoscopic ureterolithotomy; **RCT** = Randomized controlled trial; **NA** = not available; **KUB** = kidney, ureteral and bladder x-ray; **USG** = ultrasound; **CT** = computed tomography

* Jadad Quality Scale for RCT studies (score from 0 to 5)

URS and LU were indicated for large, >10mm, proximal ureteral stone in all studies. Most of the studies performed semi-rigid URS with laser as energy source (17, 19–22), although pneumatic lithotripsy was done in two studies (17, 18). LU was performed through retroperitoneal access in 3 studies (19, 20, 22), transperitoneal access in 2 studies (17, 21), or both in 1 study (18). Double J stent was routinely left in all patients regardless the surgical approach in most of studies (19–22). In only two studies double J stent was placed according to surgeon description (17, 18). Postoperative imaging exam as control for residual stones was different among the studies: KUB plus ultrasound was done in 1 study (17), only KUB was done in 2 studies (19, 20), KUB or computed tomography (CT) scan was done in 1 study, while CT scan alone was done in 1 study (21). One study did not report the imaging exam used after the procedure to assess stone fragments (22) (Table-2). Stone-free was considered as absence of residual fragments in 3 studies (17–19), residual fragments ≤3 in 2 studies (20, 21) and it was not clear in 1 study (22).

Tables-3 and 4 summarize the outcomes of each study included in this meta-analysis.

**Table 3:** Outcomes: operative time, length or hospital stay, and complications.

Study	Operative time (min)		LOS (days)		Complications (n)		Minor Complications (n)		Major Complications (n)*		LU conversions to open
	URS	LU	URS	LU	URS	LU	URS	LU	URS	LU	
Basiri et al.	42.7 ± 17.9	127.8 ± 41.8	0.53 ± 0.12	5.8 ± 2.3	0	11	0	8	0	3	2
Lopes Neto et al.	72.8 ± 42.0	215.0 ± 89.0	1.15 ± 0.55	3.15 ± 1.43	3	0	2	0	1	0	1
Fang et al.	49.0 ± 8.0	41.8 ± 8.0	2.8 ± 1.3	2.9 ± 0.8	0	0	0	0	0	0	0
Shao et al.	48.5 ± 7.7	65.6 ± 8.8	2.8 ± 0.6	4.9 ± 0.7	88	116	84	116	4	0	1
Kumar et al.	47.3 ± 8.2	49.1 ± 9.2	2.1 ± 0.6	2.2 ± 0.7	18	12	18	12	0	0	5
Liu et al.	61.1 ± 17.8	87.9 ± 18.3	5.1 ± 0.6	4.5 ± 0.48	5	3	3	3	2	0	0

**URS** = ureteroscopic lithotripsy; **LU** = laparoscopic ureterolithotomy; **LOS** = length of hospital stay

* Major complication = re-operation, sepsis + intensive care unit, ureteral stenosis (Clavien ≥3)

**Table 4:** Outcomes: stone-free rates and auxiliary procedures.

Study	Initial stone-free rate (n;%)		Final stone-free rate (n;%)		Auxiliary procedures (n;%)	
	URS	LU	URS	LU	URS	LU
Basin et al.	28 (56%)	44 (88%)	38 (76%)	45 (90%)	11 (22%)	5 (10%)
Lopes Neto et al.	8 (50%)	14 (93.3%)	10 (62.5%)	14 (93.3%)	2 (12.5%)	0
Fang et al.	22 (88%)	25 (100%)	25 (100%)	25 (100%)	3 (12%)	0
Shao et al.	NA	NA	125 (89.9%)	132 (97.0%)	14 (10.3%)	4 (2.9%)
Kumar et al.	NA	NA	28 (56%)	50 (100%)	13 (26%)	0
Liu et al.	23 (51.1%)	42 (93.3%)	37 (82.2%)	45 (100%)	17 (37.8%)	0

**URS** = ureteroscopic lithotripsy; **LU** = laparoscopic ureterolithotomy **NA** = not available

### Outcomes

**Figure 2:**
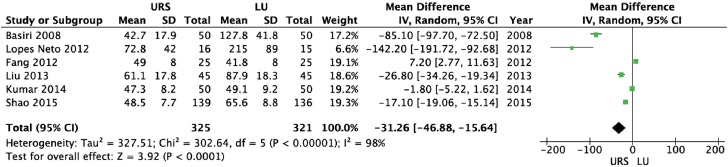
Forest plot of operative time (min).

**Figure 3:**
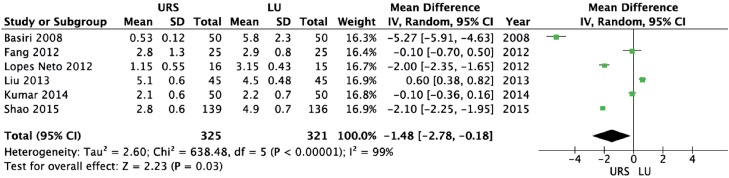
Forest plot of length of hospital stay (days).

**Figure 4:**
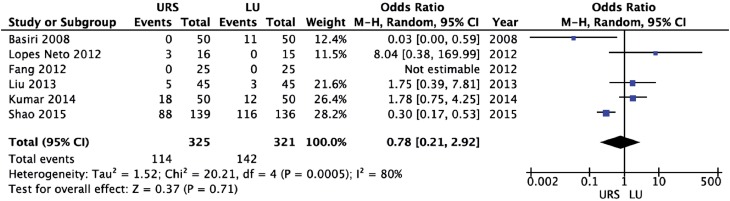
Forest plot of overall postoperative complications.

**Figure 5:**
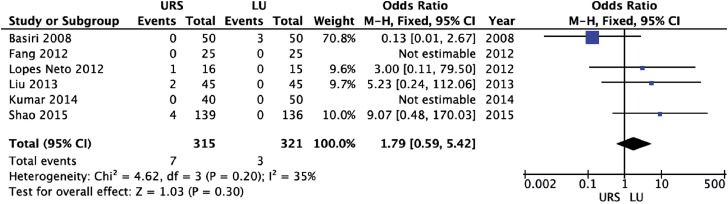
Forest plot of major postoperative complications.

URS provided a significantly shorter operative time (weighted mean difference [WMD] = −31.26 min; 95% CI-46.88 to −15.64; p<0.0001) and length of hospital stay (WMD = −1.48 days; 95% CI-2.78 to −0.18; p=0.03) than LU (Figures 2 and 3, respectively). There were no significant differences between URS and LU in terms of overall complications (OR = 0.78; 95% CI 0.21 to 2.92; p=0.71) and major complications - Clavien ≥3 - (OR = 1.79; 95% CI 0.59 to 5.42; p=0.30) (Figures 4 and 5, respectively) (15). Most of complications were minor; major complications were reported as re-operation, sepsis with need for intensive care unit, and ureteral stenosis. There were 8 conversions in the LU cases to open surgery due to technical difficulties.

**Figure 6:**
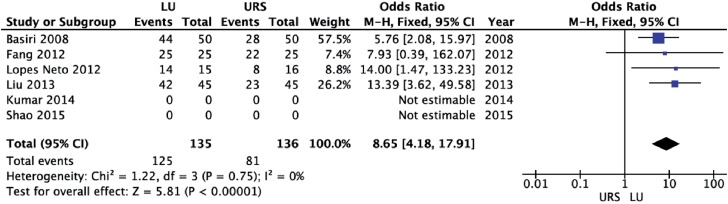
Forest plot of initial stone-free rate.

**Figure 7:**
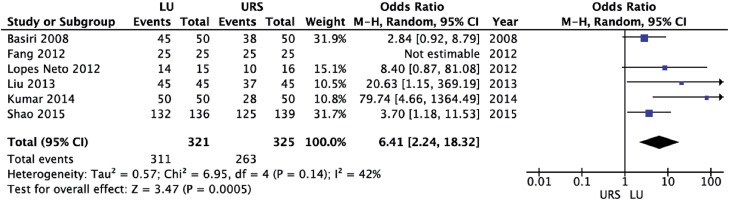
Forest plot of final stone-free rate.

**Figure 8:**
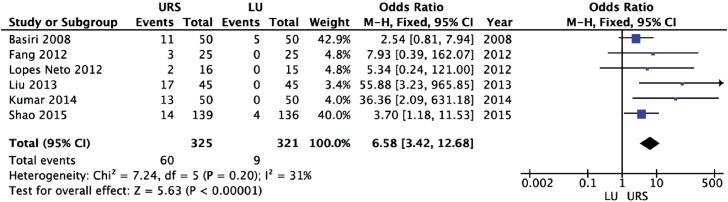
Forest plot of need for auxiliary procedures.

LU led to a significantly higher initial stone-free rate (OR = 8.65; 95% CI 4.18 to 17.91; p<0.00001) and final stone-free rate (OR = 6.41; 95% CI 2.24 to 18.32; p=0.0005) than URS (Figures 6 and 7, respectively). There was a significantly higher need for auxiliary procedures in URS cases (OR = 6.58; 95% CI 3.42 to 12.68; p<0.00001) than in LU cases (Figure-8).

## DISCUSSION

### Interpretation of data

URS has proved to be first choice of urologists, particularly young urologists, while LU has gained some popularity in the management of stones of the upper urinary tract (24–26). These findings led us to search for the current literature available comparing URS with LU in terms of peri- and postoperative outcomes. To best of our knowledge there is no meta-analysis of RCT (level of evidence 1a) regarding this relevant issue. Though semi-rigid URS is the primary modality utilized around the world, the use of flexible URS has expanded (27). Unfortunately, only one RCT including flexible URS was identified in our search, which does not provide sufficient data for a detailed evaluation. In this study, 151 patients with ureteral stones between 1 and 2cm were randomized in 3 groups (52 SWL, 51 LU and 48 flexible URS). The success rates were 96%, 81% and 79% in the laparoscopy, SWL, and URS groups, respectively. The complication rates were 7.86%, 7.06%, and 4.11% in the laparoscopy, SWL, and URS groups, respectively. While the success rate was significantly higher in the laparoscopy group (p<0.05), the complication rate was significantly lower in the URS group (p<0.05) (12).

A shorter operative time with URS was reported in five of six RCT (17, 18, 20–22), which can reflect the regular practice and the familiarity of most of urologists with this procedure. In a similar way, a shorter length of hospital stay with URS was also reported in five of six RCT (17–19, 21, 22) suggesting its less invasive nature when compared to LU leads to shorter recuperation. Regarding postoperative complications, although LU is a more invasive procedure, the risk of complications, including severe complications (Cavien ≥3) are similar. Most of complications were mild (Clavien 1 or 2), such as pain, temporary fever, and urinary tract infection. Urinary leaking was a postoperative event described more commonly after LU, but in few cases required a surgical intervention. Major complications were rare (7 cases in the URS group and 3 cases in the LU group) and were mostly re-operation due to urinary fistula or late ureteral stenosis. These data show the low morbidity of both URS and LU procedures and probably reflect patient's characteristics, stone disease features, and surgeon's experience with URS and LU of each study. With regards to BMI impact on surgical outcomes, none study included in the meta-analysis reported and/or compared the BMI between the groups, preventing us of performing any comment about that. URS can be safely and equally performed in normal, obese, and morbid obese patients (28). To best our knowledge, there is paper no evaluating the impact of obesity / BMI on LU.

Another variable that should be taken into account when evaluating the complication rate from LU is if it was performed by transperitoneal or retroperitoneal way. There is one randomized comparison study, including 48 patients that compared transperitoneal or retroperitoneal LU. The stone-free rate was similar between the groups, however transperitoneal LU was significantly associated with more pain, ileus, and longer hospital stay than retroperitoneal LU (29). In our study, most of LU was done by retroperitoneal access (211 of 321 cases), which may have contributed for the low complication rate.

Removing the stone and relieving the pain are the main purposes of URS and LU. Initial and final stone-free rates were higher with LU in all studies, showing its high efficiency (17–22). There was a higher initial (8-fold) and final (6-fold) stone-free rate with LU. The inferiority of URS may be related to the difficulty of reaching the proximal ureter with semi-rigid scopes, as flexible ureteroscopes were not used in these RCT. Furthermore, dusting the stones can lead to stone migration to the kidney, impacting negatively on stone-free rates. Another factor that needs be taken into account is the source of energy used to stone fragmentation during URS. Two of six studies used pneumatic lithotripter instead of laser to break the stone (17, 18) which is not the gold standard and may have influenced the surgical outcomes (30, 31). However, these studies reported similar final stone-free rates when compared to the studies that utilized laser as energy source. Lastly, it is important to note that the imaging modality used to evaluate postoperative stone-free rates was not the same in all studies, varying from KUB to CT scan, which have different accuracies for residual fragments (32, 33).

The need for auxiliary procedures followed the initial stone-free rate. As it was lower with URS, auxiliary procedure had a higher indication in all studies (17–22). The most common auxiliary procedure was SWL. There was a 7-fold higher risk of need for auxiliary procedures with URS than LU.

Heterogeneity among studies was found to be high for several parameters. Difference in surgical practices, follow-up imaging exams, and outcomes definitions may explain that. Despite this heterogeneity, this meta-analysis of RCT provides strong evidence (level 1a) when comparing intra- and postoperative outcomes from URS and LU. It may help urologists when choosing between these procedures for treatment of large proximal ureteral stones, mainly young urologists that have expertise with both techniques. The main limitation of this study is that flexible ureteroscopy was not take into account, but there are few well-designed studies comparing flexible ureteroscopy to laparoscopy in the management of ureteral stones, preventing us of performing a systematic evaluation.

## CONCLUSIONS

Meta-analysis of RCT suggests that LU provides a higher stone-free rate than URS in the management of large proximal ureteral stones. There are no differences regarding overall postoperative complications or major postoperative complications between the procedures. Semi-rigid URS is associated with a short operative time and length of hospital stay, however it leads to a higher need for auxiliary procedures. When counseling a patient with a large proximal ureteral stone, LU should be advised as the procedure with the higher chance of stone removal, although it is also more invasive, leading to longer operative time and length of hospital stay. Utilization of flexible ureteroscopy in conjunction with semi-rigid ureteroscopy may impact these outcomes, and deserves further systematic evaluation.
